# Characteristics of Patients Diagnosed With Guillain-Barré Syndrome at King Abdulaziz University Hospital, Jeddah, Saudi Arabia, From 2000 to 2018

**DOI:** 10.7759/cureus.48703

**Published:** 2023-11-12

**Authors:** Shahad Almalki, Lama Alghamdi, Jumana Khayyat, Rawan T Harun, Mayar Alyousef, Rana Hakeem, Sarah Alsamiri, Zienab Alrefaie, Ahmed K Bamaga

**Affiliations:** 1 Department of Neurology, Faculty of Medicine, King Abdulaziz University, Jeddah, SAU; 2 Department of Physiology, Faculty of Medicine, King Abdulaziz University, Jeddah, SAU; 3 Department of Pediatric Neurology, Faculty of Medicine, King Abdulaziz University, Jeddah, SAU

**Keywords:** prognostic factor, paralysis, myelin sheath, epidemiology study, acute inflammatory demyelinating polyneuropathy, guillain–barré syndrome

## Abstract

Background: Guillain-Barré syndrome (GBS) is the leading cause of non-polio acute flaccid paralysis worldwide, emphasizing the importance of epidemiological studies on this condition. Therefore, well-designed epidemiological studies in different populations can provide a better understanding of the characteristics of patients with GBS and the nature of the disease. To our knowledge, no previous study has attempted to describe the characteristics of patients with GBS in Kingdom of Saudi Arabia (KSA) based on disease subtypes and clinical features in both adult and pediatric patients. This study aimed to assess the frequencies of GBS subtypes and their relationships with patient characteristics and clinical data in a tertiary hospital in Jeddah, KSA.

Methods: This was a retrospective review of patients diagnosed with GBS between January 2000 and January 2018 at King Abdulaziz University Hospital (KAUH), a tertiary center in Jeddah, KSA.

Results: In total, 47 patients with GBS (median age: seven years for pediatric and 36 years for adult patients) were included in the current study. There were six male and three female pediatric patients and 19 male and 19 female adult patients. Among patients with GBS who were classified into a specific electrophysiological subtype (n = 28), 13 (46.2%) had acute inflammatory demyelinating polyneuropathy (AIDP), 11 (39%) had an axonal subtype, and four (14%) had Miller Fisher syndrome (MFS). Patients required prolonged hospitalization of approximately 20 ± 22 days (2.83 ± 3.11 weeks). Patients with MFS were more likely to have higher cytoalbuminologic dissociation than those with other subtypes.

Conclusion: AIDP was the most frequent type of GBS, followed by the axonal type. Patients required prolonged hospitalization of approximately 20 ± 22 days (2.83 ± 3.11 weeks). Patients with MFS were more likely to have higher cytoalbuminologic dissociation than those with other subtypes. GBS type did not show a relationship with ICU admission or mechanical ventilation use. There was no association between specific therapies and different GBS subtypes and no significant difference in outcomes between different patterns of clinical presentation. Intravenous immunoglobulin (IVIg) and plasma exchange (PE) treatments both had the same efficacy in relation to outcomes for patients with GBS.

## Introduction

Guillain-Barré syndrome (GBS) is an autoimmune polyradiculoneuropathy with an acute-onset and monophasic pattern that leads to flaccid paralysis. GBS frequently necessitates prolonged intensive care unit (ICU) stays, with at least 67% of patients (n=51) experiencing one major complication [1,2]. Although GBS was commonly perceived as a singular disorder with some variations, it is now recognized as a collection of syndromes consisting of multiple subtypes that primarily show distinctive pathologies and electrodiagnostic characteristics [3]. The underlying etiology and pathophysiology of GBS are not completely understood; however, the disease is often initiated by an immune-mediated process triggered by viral or bacterial infections, particularly *Campylobacter jejuni* infections [4,5].

GBS is a sporadic condition that is categorized according to the site of the immune attack, which may occur either on the myelin sheath or the axon itself. GBS, when presented with immune attacks targeting the myelin sheath, is classified as acute inflammatory demyelinating polyneuropathy (AIDP), whereas GBS involving an axonal attack is categorized as acute motor axonal neuropathy (AMAN), acute motor and sensory axonal neuropathy (AMSAN), or Miller Fisher syndrome (MFS) [6]. The incidence of these four major subtypes differs among geographic regions, with AIDP predominating in Western countries and AMAN being the predominant subtype in Central America, South America, Japan, and China [7,8].

GBS is the most common cause of non-polio acute flaccid paralysis worldwide, highlighting the importance of epidemiological studies of this disease [9]. In this regard, well-designed epidemiological studies in different populations can provide a better understanding of the characteristics of patients with GBS and the nature of the disease.

A systematic review and meta-analysis of the incidence of GBS published in 2011 showed an increase in the incidence from 0.62 to 2.66 per 100,000 person-years across all age groups [10]. However, these results were based on data collected from studies conducted in North America and Europe, and the values for the increase in incidence may be different if data were available from more studies conducted in other parts of the world. A previous systematic review of GBS in Arab countries highlighted an insufficiency of epidemiological data, with the only incidence data being reported from Benghazi, Libya (1.7 per 100,000 person-years) based on findings obtained in 1987 [11,12]. Furthermore, even fewer studies have attempted GBS subtype-based analyses, particularly in Arab countries. In the Kingdom of Saudi Arabia (KSA), only one retrospective study has been conducted in a tertiary hospital in Riyadh to determine the prevalence of GBS among pediatric patients [13]. In the study, 100 (49%) patients had GBS, with a mean age of 7 ± 3.7 years. Among the patients studied, 34 (69.4%) developed advanced paralysis in ≤2 weeks, whereas 15 (30.6%) showed advanced paralysis after two weeks.

To our knowledge, no previous study in KSA has attempted to describe the characteristics of patients with GBS based on disease subtypes and clinical features in both adult and pediatric patients. A careful literature review of studies published in KSA revealed that research on GBS is limited. Therefore, this study aimed to assess the frequencies of GBS subtypes and their relationships with patient characteristics and clinical data in a tertiary center in Jeddah, KSA.

## Materials and methods

Study design

This is a retrospective study of patients diagnosed with GBS between January 2000 and January 2018 at King Abdulaziz University Hospital (KAUH), a tertiary center in Jeddah, KSA. The study was approved by the institutional review board of King Abdulaziz University Hospital, Unit of Biomedical Ethics Research Committee (approval number: 479-19). Due to the retrospective nature of the study, informed consent was not required.

Study participants

Adult and pediatric patients with GBS admitted to KAUH between 2000 and 2018 were considered for the study. We used the well-known Brighton criteria to identify patients with (i) bilateral and flaccid weakness of the limbs, (ii) a monophasic illness pattern characterized by an interval of 12 hours to 28 days between the onset of symptoms and the point of maximum weakness, followed by subsequent clinical plateau, and (iii) an absence of an alternative diagnosis for the observed weakness, along with at least one of the following: (a) areflexia or hyporeflexia in the affected limbs, (b) cytoalbuminologic dissociation, defined as CSF protein levels above the normal reference values with a cell count <50 cells/µL, or (c) electrophysiological findings consistent with GBS [14,15]. Because deep tendon reflexes are normal in some patients with GBS [15], we included patients with preserved deep tendon reflexes.

Since KAUH was converting its earlier paper-based health record system to an electronic system during our study period, raw nerve conduction studies and electromyographic data to confirm the electrophysiological subtype were not available for all patients. Therefore, we used the electrophysiological subtype documented by the treating neurologist for our analyses.

We also included patients with MFS presenting with the typical triad of areflexia, ataxia, and ophthalmoplegia. Patients with GBS-MFS overlap syndrome were also included in the study.

We excluded patients with (i) acute-onset chronic inflammatory demyelinating polyneuropathy (A-CIDP), (ii) comorbid diabetic neuropathy, (iii) acute paralysis other than GBS, such as myasthenia gravis, polio, or botulism, or (iv) the onset of weakness post-bariatric surgery.

Data collection and variables

A Google Form (Google LLC, Mountain View, California, United States) consisting of three main sections was used to collect data from the records. The first section evaluated demographic information and information regarding antecedent infections. The second section evaluated the presence of the following clinical features upon presentation: distribution of weakness, deep tendon reflex status, ataxia, sensory loss, cranial nerve involvement, and autonomic and sphincter involvement. The third section assessed the hospital course and follow-up, investigation results (CSF analysis and electrophysiological studies), therapy administered, ICU admission, mechanical ventilation, duration of hospitalization, and recovery at follow-up.

The first set of results was obtained from patients who underwent multiple electrophysiological studies during the same period of diagnosis. Electrophysiological subtypes were labeled as AIDP, axonal variant (AMSAN or AMAN), MFS, or unclassified owing to equivocal results or absence of electrophysiological studies because they were not requested by the treating neurologist.

Statistical analysis

The data were analyzed using IBM SPSS Statistics for Windows, Version 24.0 (Released 2016; IBM Corp., Armonk, New York, United States). Categorical data were analyzed using the chi-squared or likelihood-ratio chi-squared test when the expected count was <5. Continuous data are reported as mean and standard deviation (SD) or median and quartiles. As the variables were not normally distributed, the non-parametric Kruskal-Wallis test was performed to assess differences among them. Statistical significance was set at a two-sided P < 0.05.

## Results

A total of 47 patients with GBS (median age: seven years for pediatric patients and 36 years for adult patients) were included in the current study. There were six male and three female pediatric patients and 19 male and 19 female adult patients. Among the patients with GBS who were classified into a specific electrophysiological subtype (n = 28), 13 (46.2%) had AIDP, 11 (39%) had an axonal subtype, and four (14%) had MFS. The demographic characteristics, investigation results, therapy-related data, and outcomes of the patients with GBS are shown in Table 1.

**Table 1:** Demographic characteristics, electrophysiological subtypes, investigation results, and therapy- and outcome-related data of patients with GBS AIDP, acute inflammatory demyelinating polyneuropathy; AMAN, acute motor axonal neuropathy; AMSAN, acute motor and sensory axonal neuropathy; CSF, cerebrospinal fluid; GBS, Guillain-Barré syndrome; GI, gastrointestinal; ICU, intensive care unit; IQR, interquartile range; IVIg, intravenous immunoglobulin; SD, standard deviation; URTI, upper respiratory tract infection. *Cytoalbuminologic dissociation: defined as elevated CSF protein level with CSF cell count less than 50 cells/µL according to the Brighton criteria. **Due to the lack of nerve conduction study raw data/report. ***Seven patients received IVIg + plasma exchange + physiotherapy, while one patient received IVIg + plasma exchange. The data has been represented as N, %, mean ±SD.

Variable	n (%)
Age at diagnosis, years	Mean (SD)
Pediatric	7.6 (4.4)
Adult	38.8 (18.7)
Sex	n (%)
Male	25/47 (53.2%)
Female	22/47 (46.8%)
Antecedent infection	23/47 (48.9%)
URTI (fever, runny nose)	21/23 (91.3%)
GI tract infection (diarrhea, abdominal pain)	1/23 (4.3%)
Both	1/23 (4.3%)
CSF analysis	26/47 (55.3%)
Normal results	7/26 (26.9%)
High glucose level	15/26 (57.7%)
Cell count > 50 cell/µL	3/26 (11.5%)
Elevated CSF protein with cell count > 50 cell/µL	3/26 (11.5%)
Cytoalbuminologic dissociation*	5/26 (19.2%)
Electrophysiological subtypes	28/47 (59.6%)
AIDP	13/28 (46.4%)
Axonal	11/28 (39.3%)
AMAN	4/11 (36.4%)
AMSAN	3/11 (27.3%)
Miller Fisher syndrome	4/28 (14.3%)
Unclassified**	19/47 (40.4%)
Therapy	45/47 (95.7%)
IVIg only	33/ 45 (73.3%)
Plasma exchange only	1/45 (2.2%)
Physiotherapy only	3/45 (6.7%)
Combination therapy***	8/45 (17.8%)
ICU admission	18 /47 (38.3%)
Mechanical ventilation/Intubation	16/47 (34.04%)
Recovery/Outcome at follow-up	
Full recovery	16/47 (34.04%)
Partial recovery	25/47 (53.2%)
No improvement	2/47 (4.3%)
Discharged against medical advice	3/47 (6.4%)
Deceased	1/47 (2.1%)
Duration of hospitalization	Mean (SD)
Days	20 (22)
Weeks	2.83 (3.11)

Clinical features at presentation

The clinical presentations of the patients with GBS at disease onset are shown in Table 2. Motor weakness was observed in most patients with GBS (n=46; 98%). Of them, 26 (79%) had greater weakness in the lower limbs than in the upper limbs. Greater distal-than-proximal weakness was reported in 34 (72%) patients. Sensory loss was also reported in 28 (60%) patients, mainly in the form of paresthesia/numbness (n=23; 82%).

**Table 2:** Clinical presentation of patients with GBS at disease onset GBS, Guillain–Barré syndrome The data has been represented as N,%, mean ±SD

Variable	n (%)
Motor weakness pattern	46/47 (97.9%)
Generalized weakness pattern	33/46 (70.2%)
Lower limb > upper limb	26/33 (78.8%)
Upper limb > lower limb	5/33 (15.2%)
Upper limb = lower limb	2/33 (6.1%)
Lower limb-only weakness	11/46 (23.4%)
Upper limb-only weakness	2/46 (4.3%)
Weakness pattern within the limb	
Distal weakness > proximal weakness	34/47 (72.3%)
Proximal weakness > distal weakness	9/47 (19.1%)
Proximal weakness = distal weakness	3/47 (6.4%)
Absent or diminished deep tendon reflexes	17/47 (36.2%)
Ataxia	2/47 (4.3%)
Sensory loss	28/47 (59.6%)
Paresthesia/numbness	23/28 (82.1%)
Loss of pain	5/28 (17.9%)
Loss of touch	4/28 (14.3%)
Loss of proprioception	3/28 (10.7%)
Gloves-stocks loss of sensation	1/28 (3.6%)
Cranial nerve involvement	24/47 (51.1%)
Ophthalmoplegia	14/24 (58.3%)
Facial palsy	15/24 (62.5%)
Oropharyngeal weakness	17/24 (70.8%)
Autonomic involvement	13/47 (27.7%)
Blood pressure and heart rate dysregulation	4/13 (30.8%)
Intestinal dysfunction	4/13 (30.8%)
Voiding dysfunction	7/13 (53.8%)

Comparisons among GBS subtypes

The investigation results and hospital courses for the different GBS subtypes are shown in Table 3. The three electrophysiological subtypes showed no differences in age at diagnosis, occurrence of antecedent infection, type of therapy administered, ICU admission, need for mechanical ventilation or intubation, recovery and improvement rates, or duration of hospitalization (P > 0.05). However, GBS subtypes were associated with CSF analysis results, as two (67%) patients with MFS showed cytoalbuminologic dissociation compared with 0% of patients with the AIDP and axonal subtypes (P < 0.05, chi-squared test).

**Table 3:** Investigation results and hospital courses associated with different GBS subtypes AIDP, acute inflammatory demyelinating polyneuropathy; CSF, cerebrospinal fluid; GBS, Guillain–Barré syndrome; GI, gastrointestinal; ICU, intensive care unit; IQR, interquartile range; IVIg, intravenous immunoglobulin; SD, standard deviation; URTI, upper respiratory tract infection. P < 0.05 was considered significant. The data has been represented as N, %, mean ±SD, and chi-squared test.

	GBS subtypes, n (%)			
	AIDP	Axonal	Miller Fisher syndrome	p-value
Age at diagnosis, mean (SD)	39.2 (25.4)	33.1 (16.8)	31.8 (20.2)	0.805
Antecedent infection, n (N%)				
No infection	7/13 (53.8%)	5/11 (45.5%)	1/4 (25%)	0.658
Both	1/13 (7.7%)	0/11 (0%)	0/4 (0%)	
GI tract infection	0/13 (0%)	1/11 (9.1%)	0/4 (0%)	
URTI	5/13 (38.5%)	5/11 (45.5%)	3/4 (75%)	
Therapy, n (N%)				
Combination therapy	2/13 (15.4%)	4/11 (36.4%)	0/4 (0%)	0.323
IVIg only	9/13 (69.2%)	6/11 (54.5%)	4/4 (100%)	
Physiotherapy only	2/13 (15.4%)	0/11 (0%)	0/4 (0%)	
Plasma exchange only	0/13 (0%)	1/11 (9.1%)	0/4 (0%)	
CSF analysis				
Normal results	1/5 (20%)	2/5 (40%)	1/3 (33.3%)	0.781
Cell count > 50 cells/µL	2/5 (40%)	1/5 (20%)	0/3 (0%)	0.315
High glucose level	3/5 (60%)	2/5 (40%)	2/3 (66.7%)	0.717
Cytoalbuminologic dissociation	0/5 (0%)	0/5 (0%)	2/3 (66.7%)	0.025
Elevated CSF protein level	0/5 (0%)	0/5 (0%)	0/3 (0%)	--
ICU admission, n (N%)	6/13 (46.2%)	6/11 (54.5%)	2/4 (50%)	0.92
Mechanical ventilation/Intubation, n (N%)	5/13 (38.5%)	7/11 (63.6%)	2/4 (50%)	0.47
Recovery/outcome at follow-up, n (N%)				
Full recovery	4/13 (30.8%)	4/11 (36.4%)	1/4 (25%)	0.415
No improvement	0/13 (0%)	2/11 (18.2%)	0/4 (0%)	
Partial recovery	9/13 (69.2%)	5/11 (45.5%)	3/4 (75%)	
Duration of hospitalization, Mean (SD)	11 (31)	28 (60)	14.5 (23)	0.266

The clinical presentations of patients with different GBS subtypes are shown in Table 4. The three electrophysiological subtypes showed no significant differences in motor weakness patterns or deep tendon reflexes (P > 0.05). However, the presence of sensory loss was associated with the GBS subtype, as three (75%) patients with MFS showed no sensory loss compared with six (46%) and one (9%) patients with the AIDP and axonal subtypes, respectively (P < 0.05, chi-squared test). Cranial nerve involvement was also associated with the GBS subtype. All (100%) patients with MFS had ophthalmoplegia compared with four (31%) and three (27%) patients with the AIDP and axonal subtypes, respectively (P < 0.05, chi-squared test). Furthermore, autonomic involvement was associated with the GBS subtype, as it was absent in all (100%) patients with MFS and 12 (92%) patients with AIDP, whereas it was absent in five (46%) patients with the axonal subtype (P < 0.05, chi-squared test).

**Table 4:** Clinical presentation of patients with different GBS subtypes AIDP, acute inflammatory demyelinating polyneuropathy; GBS, Guillain–Barré syndrome. P < 0.05 was considered significant. The data has been represented as N, %, mean ±SD, and chi-squared test.

		GBS subtypes, n (%)					
		AIDP		Axonal		Miller Fisher syndrome	p-value
Motor weakness pattern							
Generalized weakness		12/13 (92.3%)		8/11 (72.7%)		2/4 (50%)	0.099
Lower limb-only weakness		1/13 (7.7%)		2/11 (18.2%)		0/4 (0%)	
No limb weakness		0/13 (0%)		0/11 (0%)		1/4 (25%)	
Upper limb-only weakness		0/13 (0%)		1/11 (9.1%)		1/4 (25%)	
Absent or diminished deep tendon reflexes							
No		9/13 (69.2%)		5/11 (45.5%)		1/4 (25%)	0.236
Yes		4/13 (30.8%)		6/11 (54.5%)		3/4 (75%)	
Weakness pattern within the limb							
Distal weakness > proximal weakness		10/13 (76.9%)		8/11 (72.7%)		1/4 (25%)	0.113
No limb weakness		0/13 (0%)		0/11 (0%)		1/4 (25%)	
Proximal weakness = distal weakness		1/13 (7.7%)		0/11 (0%)		1/4 (25%)	
Proximal weakness > distal weakness		2/13 (15.4%)		3/11 (27.3%)		1/4 (25%)	
Generalized weakness pattern							
Lower limb weakness > upper limb weakness		9/13 (69.2%)		7/11 (63.6%)		2/4 (50%)	0.46
No generalized weakness		1/13 (7.7%)		3/11 (27.3%)		2/4 (50%)	
Upper limb weakness = lower limb weakness		2/13 (15.4%)		0/11 (0%)		0/4 (0%)	
Upper limb weakness > lower limb weakness		1/13 (7.7%)		1/11 (9.1%)		0/4 (0%)	
Sensory loss							
Paresthesia/numbness		7/13 (53.8%)		7/11 (63.6%)		1/4 (25%)	0.415
Loss of proprioception		1/13 (7.7%)		1/11 (9.1%)		0/4 (0%)	0.828
Loss of pain		1/13 (7.7%)		2/11 (18.2%)		0/4 (0%)	0.537
Loss of touch		0/13 (0%)		2/11 (18.2%)		0/4 (0%)	0.189
Gloves-stocks loss of sensation		0/13 (0%)		1/11 (9.1%)		0/4 (0%)	0.449
No sensory loss		6/13 (46.2%)		1/11 (9.1%)		3/4 (75%)	0.035
Cranial nerve involvement							
No		6/13 (46.2%)		5/11 (45.5%)		0/4 (0%)	0.221
Facial palsy		6/13 (46.2%)		4/11 (36.4%)		2/4 (50%)	0.848
Oropharyngeal weakness		5/13 (38.5%)		4/11 (36.4%)		3/4 (75%)	0.372
Ophthalmoplegia		4/13 (30.8%)		3/11 (27.3%)		4/4 (100%)	0.027
Autonomic involvement							
No autonomic involvement		12/13 (92.3%)		5/11 (45.5%)		4/4 (100%)	0.014
Blood pressure and/or heart rate dysregulation		0/13 (0%)		2/11 (18.2%)		0/4 (0%)	0.189
Voiding dysfunction		1/13 (7.7%)		3/11 (27.3%)		0/4 (0%)	0.267
Intestinal dysfunction		0/13 (0%)		2/11 (18.2%)		0/4 (0%)	0.189
Sphincter involvement		1/13 (7.7%)		2/11 (18.2%)		0/4 (0%)	0.537

Comparison of weakness patterns

Patterns of motor weakness were not associated with antecedent infections (Table 5). However, they were significantly associated with CSF results, as one (100%) patient with no limb weakness showed normal CSF results compared with two (12%) with generalized weakness (P < 0.05, chi-squared test) (Table 6). In addition, 13 (77%) patients with generalized weakness had high glucose levels compared with two (29%) patients with lower limb-only weakness and 0% with upper limb-only or no limb weakness (P < 0.05, chi-squared test). In contrast, motor weakness patterns showed no associations with ICU admission, duration of hospitalization, or improved outcomes at follow-up (Tables 7, 8).

**Table 5:** Association between motor weakness patterns and antecedent infection GBS, Guillain–Barré syndrome; GI, gastrointestinal; URTI, upper respiratory tract infection. P < 0.05 was considered significant. The data has been represented as N, %, mean ±SD, and chi-squared test.

		Antecedent infection, n (%)	
		GI tract	
	No infection	infection	URTI
Motor weakness pattern			
No limb weakness (n = 1)	0/1 (0%)	0/1 (0%)	1/1 (100%)
Generalized weakness (n = 33)	17/33 (51.5%)	2/33 (6.1%)	15/33 (45.5%)
Lower limb-only weakness (n = 11)	6/11 (54.5%)	0/11 (0%)	5/11 (45.5%)
Upper limb-only weakness (n = 2)	1/2 (50%)	0/2 (0%)	1/2 (50%)
p-value	0.685	0.693	0.669
Generalized weakness pattern			
No generalized weakness (n = 14)	7/14 (50%)	0/14 (0%)	7/14 (50%)
Lower limb weakness > upper limb weakness (n = 5)	2/5 (40%)	0/5 (0%)	3/5 (60%)
Upper limb weakness = lower limb weakness (n = 2)	2/2 (100%)	0/2 (0%)	0/2 (0%)
Upper limb weakness > lower limb weakness (n = 5)	3/5 (60%)	1/5 (20%)	1/5 (20%)
p-value	0.388	0.324	0.232
Weakness pattern within the limb			
No limb weakness (n = 1)	0/1 (0%)	0/1 (0%)	1/1 (100%)
Distal weakness > proximal weakness (n = 34)	19/34 (55.9%)	1/34 (2.9%)	15/34 (44.1%)
Proximal weakness = distal weakness (n = 3)	1/3 (33.3%)	0/3 (0%)	2/3 (66.7%)
Proximal weakness > distal weakness (n = 9)	4/9 (44.4%)	1/9 (11.1%)	4/9 (44.4%)
p-value	0.515	0.744	0.548

**Table 6:** Association between motor weakness patterns and CSF analysis results CSF, cerebrospinal fluid. P < 0.05 was considered significant The data has been represented as N, %, mean ±SD, and chi-squared test.

	CSF analysis, n (%)						
	Normal results	Cell count > 50 cells/µL	High glucose	Cytoalbuminologic dissociation		Elevated CSF protein	
Motor weakness pattern							
No limb weakness (n = 1)	1/1 (100%)	0/1 (0%)	0/1 (0%)	0/1 (0%)	0/1 (0%)		
Generalized weakness (n = 17)	2/17 (11.8%)	3/17 (17.6%)	13/17 (76.5%)	5/17 (29.4%)	1/17 (5.9%)		
Lower limb-only weakness (n = 7)	4/7 (57.1%)	2/7 (28.6%)	2/7 (28.6%)	0/7 (0%)	2/7 (28.6%)		
Upper limb-only weakness (n = 1)	0/1 (0%)	1/1 (100%)	0/1 (0%)	0/1 (0%)	0/1 (0%)		
p-value	0.038	0.276	0.037	0.182	0.285		
Generalized weakness pattern							
No generalized weakness (n = 9)	5/9 (55.6%)	3/9 (33.3%)	2/9 (22.2%)	0/9 (0%)	2/9 (22.2%)		
Lower limb weakness > upper limb weakness (n = 4)	0/4 (0%)	1/4 (25%)	3/4 (75%)	2/4 (50%)	0/4 (0%)		
Upper limb weakness > lower limb weakness (n = 1)	0/1 (0%)	0/1 (0%)	1/1 (100%)	0/1 (0%)	0/1 (0%)		
p-value	0.053	0.672	0.079	0.051	0.377		
Weakness pattern within the limb							
No limb weakness (n = 1)	1/1 (100%)	0/1 (0%)	0/1 (0%)	0/1 (0%)	0/1 (0%)		
Distal weakness > proximal weakness (n = 17)	5/17 (29.4%)	3/17 (17.6%)	11/17 (64.7%)	3/17 (17.6%)	2/17 (11.8%)		
Proximal weakness = distal weakness (n = 2)	0/2 (0%)	0/2 (0%)	2/2 (100%)	0/2 (0%)	0/2 (0%)		
Proximal weakness > distal weakness (n = 6)	1/6 (16.7%)	3/6 (50%)	2/6 (33.3%)	2/6 (33.3%)	1/6 (16.7%)		
p-value	0.232	0.269	0.126	0.578	0.832		

**Table 7:** Association between motor weakness patterns and hospital course ICU, intensive care unit. P < 0.05 was considered significant. The data has been represented as N, %, mean ±SD, and chi-squared test.

	Mechanical ventilation/Intubation n (%)	ICU admission n (%)	Duration of hospitalization Mean (SD)
Motor weakness pattern			
Generalized weakness (n = 33)	13/33 (39.4%)	15/33 (45.5%)	14 (22)
Lower limb-only weakness (n = 11)_	2/11 (18.2%)	2/11 (18.2%)	7 (9)
No limb weakness (n = 1)	0/1 (0%)	0/1 (0%)	7
Upper limb-only weakness (n = 2)	1/2 (50%)	1/2 (50%)	26
p-value	0.494	0.343	0.237
Generalized weakness pattern			
Lower limb weakness > upper limb weakness (n = 26)	12/26 (46.2%)	13/26 (50%)	16.5 (25)
No generalized weakness (n = 14)	3/14 (21.4%)	3/14 (21.4%)	7 (15)
Upper limb weakness = lower limb weakness (n = 2)	1/2 (50%)	1/2 (50%)	6
Upper limb weakness > lower limb weakness (n = 5)	0/5 (0%)	1/5 (20%)	12 (10)
p-value	0.139	0.26	0.206
Weakness pattern within the limb			
Distal weakness > proximal weakness (n = 34)	12/34 (35.3%)	13/34 (38.2%)	9 (25)
No limb weakness (n = 1)	0/1 (0%)	0/1 (0%)	7
Proximal weakness = distal weakness (n = 3)	1/3 (33.3%)	1/3 (33.3%)	14
Proximal weakness > distal weakness (n = 9)	3/9 (33.3%)	4/9 (44.4%)	22 (32)
p-value	0.909	0.85	0.438

**Table 8:** Association between motor weakness patterns and recovery/outcome at follow-up P < 0.05 was considered significant. The data has been represented as N, %, mean ±SD, and chi-squared test.

	Recovery/Outcome at follow-up, n (%)						
	Deceased		Discharged against medical advice	Full recovery		No improvement	Partial recovery
Motor weakness pattern							
Generalized weakness (n = 33)	0/33 (0%)		2/33 (6.1%)	10/33 (30.3%)		2/33 (6.1%)	19/33 (57.6%)
Lower limb-only weakness (n = 11)	1/11 (9.1%)		1/11 (9.1%)	4/11 (36.4%)		0/11 (0%)	5/11 (45.5%)
No limb weakness (n = 1)	0/1 (0%)		0/1 (0%)	1/1 (100%)		0/1 (0%)	0/1 (0%)
Upper limb-only weakness (n = 2)	0/2 (0%)		0/2 (0%)	1/2 (50%)		0/2 (0%)	1/2 (50%)
p-value	0.87						
Generalized weakness pattern							
Lower limb weakness > upper limb weakness (n = 26)	0/26 (0%)		1/26 (3.8%)	9/26 (34.6%)		1/26 (3.8%)	15/26 (57.7%)
No generalized weakness (n = 14)	1/14 (7.1%)		1/14 (7.1%)	6/14 (42.9%)		0/14 (0%)	6/14 (42.9%)
Upper limb weakness = lower limb weakness (n = 2)	0/2 (0%)		0/2 (0%)	0/2 (0%)		0/2 (0%)	2/2 (100%)
Upper limb weakness > lower limb weakness (n = 5)	0/5 (0%)		1/5 (20%)	1/5 (20%)		1/5 (20%)	2/5 (40%)
p-value	0.574						
Weakness pattern within the limb							
Distal weakness > proximal weakness (n = 34)	1/34 (2.9%)		3/34 (8.8%)	12/34 (35.3%)		1/34 (2.9%)	17/34 (50%)
No limb weakness (n = 1)	0/1 (0%)		0/1 (0%)	1/1 (100%)		0/1 (0%)	0/1 (0%)
Proximal weakness = distal weakness (n = 3)	0/3 (0%)		0/3 (0%)	1/3 (33.3%)		0/3 (0%)	2/3 (66.7%)
Proximal weakness > distal weakness (n = 9)	0/9 (0%)		0/9 (0%)	2/9 (22.2%)		1/9 (11.1%)	6/9 (66.7%)
p-value	0.939						

Therapy types and recovery or outcomes

No significant association was observed between therapy type and recovery or outcome at follow-up in patients with GBS (P > 0.05) (Table 9).

**Table 9:** Relationship between therapy type and recovery/outcome at follow-up in GBS patients IVIg, intravenous immunoglobulin; DAMA, discharged against medical advice. P < 0.05 was considered significant. The data has been represented as N, %, mean ±SD, and chi-squared test.

	Deceased	Discharged against medical advice	Full recovery	No improvement	Partial recovery
Combination therapy (n = 8)	0/8 (0%)	0/8 (0%)	3/8 (37.5%)	0/8 (0%)	5/8 (62.5%)
IVIg only (n = 33)	1/33 (3%)	1/33 (3%)	11/33 (33.3%)	2/33 (6.1%)	18/33 (54.5%)
No therapy – DAMA (n = 2)	0/2 (0%)	2/2 (100%)	0/2 (0%)	0/2 (0%)	0/2 (0%)
Physiotherapy only (n = 3)	0/3 (0%)	0/3 (0%)	1/3 (33.3%)	0/3 (0%)	2/3 (66.7%)
Plasma exchange only (n = 1) p-value	0/1 (0%) 0.367	0/1 (0%)	1/1 (100%)	0/1 (0%)	0/1 (0%)

## Discussion

Our study reviewed 47 patients with GBS and showed that the number of male patients (n=25; 53.2%) was slightly greater than the number of female patients (n=22; 46.8%), with a male-to-female ratio of 1:1 in adult patients and 2:1 in pediatric patients. Considering the supposed autoimmune etiology of GBS, the male predominance is unusual and somewhat unexplainable for an autoimmune disease [15]. This male predominance may be attributable to the protective effects of estrogen against infections and autoimmune responses [16,17]. Similar results have been obtained in different countries, such as Brazil (male-to-female ratio, 1.4/1.0) and Kuwait (male-to-female ratio, 2.7:1) [15,13]. However, in one study on childhood GBS in Kuwait, girls were reported to be more frequently affected than boys [18].

The mean ages of the adult and pediatric patients with GBS were 38.8 ± 18.7 years and 7.6 ± 4.4 years, respectively. The occurrence of GBS showed a significant relationship with advancing age, consistent with the results of previous studies in Europe [10,19-28], the United States [29,30], Australia [31], China [32], and Japan [33].

Antecedent infections

In the present study, 23 (48.9%) patients had an infection before the onset of GBS symptoms. Most cases involved upper respiratory tract infections (n=21; 91.3%), followed by gastrointestinal tract infections (n=1; 4.3%). However, 24 (51.1%) patients had no documented infectious event prior to the appearance of GBS symptoms. Antecedent infections before the onset of GBS symptoms have been reported in studies from different countries [13,15,34,35]. The predominant antecedent infections in these studies were respiratory infections, followed by gastrointestinal tract infections [15,34,35].

Clinical features at presentation

In this study, motor weakness at presentation was observed in most patients with GBS and was more common in the lower than in the upper limbs, showing a distal pattern. This finding is consistent with the results of studies conducted in KSA, Kuwait, and Turkey, where motor weakness, starting mainly in the lower limbs, was the most common initial symptom [36-39].

Sensory loss was also noted in more than half of the patients (n=28) at presentation. A study conducted in Kuwait indicated that approximately half of the patients reported sensory complaints during their initial presentation (n=23) [38]. In contrast, sensory disturbances were observed in 10% of all cases in a study in Brazil [40].

We found a significant association between CSF glucose levels and motor weakness patterns in patients with GBS. Patients with generalized weakness were more likely to have high CSF glucose levels, whereas those with lower limb, upper limb, or no limb weakness were less likely to have high CSF glucose levels. Thus, CSF results may provide valuable information regarding the severity and prognosis of GBS, particularly in relation to motor weakness patterns. A study conducted in China showed that high CSF glucose levels were associated with more severe clinical outcomes and poorer short-term prognoses in patients with GBS [41]. We did not find any relationship between CSF protein levels and GBS subtypes or weakness patterns. However, another study revealed that patients with high CSF protein levels were more likely to have a demyelinating subtype and proximal or global muscle weakness. In contrast, patients with MFS and predominantly distal weakness tended to have lower CSF protein levels [42]. Our study also did not reveal any significant associations between the patterns of motor weakness, ICU admission, and the requirement for mechanical ventilation. However, many studies have identified an association between generalized weakness and the need for mechanical ventilation [43,44]. To ensure timely and appropriate treatment, it is crucial to identify early predictors of mechanical ventilation in GBS. Previous studies have revealed that the inability to raise the elbows above the bed, rapidly progressive motor weakness, involvement of the peripheral limb and axial muscles, bulbar dysfunction, facial weakness, and cranial nerve involvement in children are risk factors for mechanical ventilation [36,45-50].

Electrophysiological subtypes

The prevalence of GBS subtypes varies considerably between geographical regions. Western countries show a higher prevalence of AIDP, whereas AMAN occurs more frequently in Asia and Central and South America [3,51]. This difference in the distribution of AMAN between geographical areas may be related to the different distributions of *C. jejuni* infections, considering the high incidence of AMAN after these infections [52]. However, our study reported AIDP as the most common subtype, with a frequency of 13 (46.4%). This finding is consistent with reports from other regions of Southwest China and Turkey, in which AIDP accounted for 97 (57%) and 73 (70.2%) patients with GBS, respectively [52,53].

The incidence of MFS (n=4; 14.3%) in the current study was similar to that observed in the Kuwait study, in which three of the 41 patients (7%) had MFS [13]. In sharp contrast, the incidence of MFS-GBS in studies conducted in Taiwan [54] and Japan [55] were 19% (n=32) and 25% (n=53), respectively. Our study also showed a correlation between MFS occurrence and cytoalbuminologic dissociation (n=2; 66.7%). In contrast, an Asian cohort study demonstrated a lower occurrence of cytoalbuminologic dissociation in patients with MFS than in those with GBS. Additionally, they identified a strong correlation between the presence of cytoalbuminologic dissociation and the timing of lumbar puncture [56].

We did not find a relationship between GBS type and ICU admission or mechanical ventilation use. However, a previous study in China suggested that axonal subtypes of GBS, particularly AMAN, are associated with a higher risk of mechanical ventilation and ICU admission than AIDP [52]. According to Sundar et al. [57], patients with axonal involvement are at a higher risk of developing respiratory failure.

Type of therapy

The main treatment modalities for GBS are plasma exchange (PE), intravenous immunoglobulin (IVIg), and a combination of both [58]. Forty-five (73.3%) patients with GBS in this study received IVIg as the initial therapy, whereas eight (17.7%) received combination therapy. Both PE and IVIg have been shown to be equally effective in improving disability, duration of mechanical ventilation, mortality, and residual disability [58,59]. However, IVIg is more convenient to administer owing to its ease of application and fewer adverse effects. This factor is particularly relevant in the pediatric age group, wherein IVIg is the preferred treatment [60]. Therefore, all pediatric patients in this study received IVIg except for one patient who received PE. A similar finding was reported in a prospective multicenter study in Germany, Switzerland, and Austria, in which 87 (91.5%) children with GBS were treated with IVIg immediately after diagnosis, and improvement was seen 13 days after the first symptoms [60].

Our study showed no association between specific therapies and different GBS subtypes. Similar findings were also obtained in two randomized clinical trials conducted in patients with GBS, which found no significant effect of treatment on GBS subtypes [61,62].

Outcomes

Our study also showed no significant difference in outcome between different patterns of clinical presentation. In contrast, in a study conducted in Iran on a pediatric population, autonomic and cranial nerve involvement was associated with poor outcomes at two months, and cranial nerve involvement was associated with a significantly poor functional outcome six months after disease onset [63]. Another study conducted in a tertiary hospital in Riyadh, KSA, showed that, two months after the onset of the disease, lasting paralysis was observed in children who experienced facial weakness and ocular deficits [14]. One study conducted in a tertiary center in Kuwait with a sample encompassing multiple age groups showed that a pattern of predominant distal weakness in the lower limb, proximal weakness in the upper limb, and autonomic disturbances were poor prognostic factors for delayed recovery [13]. We concluded that IVIg and PE treatments both had the same efficacy in relation to outcomes for our patients with GBS. Similar results have been described in a review that showed no significant differences in outcomes when compared IVIg with PE efficacy [61,62].

Duration of hospitalization

The mean duration of hospitalization in our study was 20 ± 22 days (2.83 ± 3.11 weeks). We did not find any significant relationship between GBS subtypes and clinical presentation or duration of hospitalization. These findings contrast with those obtained in a study conducted in Iran, in which patients with axonal subtypes of GBS, including AMAN and AMSAN, showed longer hospital stays than those with other subtypes [35]. We also compared our findings with those from studies in other countries, such as Iran, Turkey, and the United States. The duration of hospitalization in our study was longer than that in other countries [64,65]. The lowest duration of hospitalization was in Turkey (15 ± 19.6 days) [65], followed by Iran (12.67 ± 9.58 days) [35] and the United States (median, interquartile range: 7, 5-13 days). Additionally, the United States study reported that an increased length of stay (median, interquartile range: 14, 6-27 days) was correlated with increased mortality [65].

Limitations

Our study had some limitations, which could be mainly attributed to its retrospective nature and patients lost to follow-up, potentially because most of our patients were non-Saudi and had poor documentation and missing information in the records. As such, we could not determine if the clinical presentations of patients correlated with prognosis, clinical GBS scores, or long-term outcomes. Moreover, the epidemiology of GBS may vary globally, and our small sample may not apply to all regions in KSA. Therefore, we recommend additional studies with larger populations to assess the effects of potential risk factors and provide a better understanding of patient characteristics, long-term outcomes, and prognosis in GBS, thereby facilitating the development of a universal treatment guide.

## Conclusions

Our findings provide a rough estimation of GBS background, frequency, and subtypes at KAUH. AIDP was the most frequent type, with a frequency of 46.4%, followed by the axonal type. Patients required prolonged hospitalization of approximately 20 ± 22 days (2.83 ± 3.11 weeks). Patients with MFS were likelier to have higher cytoalbuminologic dissociation than those with other subtypes. We did not find a relationship between GBS type and ICU admission or mechanical ventilation use. Our study showed no association between specific therapies and different GBS subtypes and no significant difference in outcome between different patterns of clinical presentation. We concluded that IVIg and PE treatments both had the same efficacy in relation to outcomes for our patients with GBS.
